# 4-Sodium phenyl butyric acid has both efficacy and counter-indicative effects in the treatment of Col4a1 disease

**DOI:** 10.1093/hmg/ddy369

**Published:** 2018-10-22

**Authors:** Frances E Jones, Lydia S Murray, Sarah McNeilly, Afshan Dean, Alisha Aman, Yinhui Lu, Nija Nikolova, Ruben Malomgré, Karen Horsburgh, William M Holmes, Karl E Kadler, Tom Van Agtmael

**Affiliations:** 1 Institute of Cardiovascular and Medical Sciences, College of Medical, Veterinary and Life Sciences, University of Glasgow, Glasgow, UK; 2 Wellcome Centre for Cell-Matrix Research, Faculty of Biology, Medicine & Health, University of Manchester, Manchester, UK; 3 Centre for Discovery Brain Sciences, Medical School, University of Edinburgh, Edinburgh, UK; 4 Institute of Neuroscience and Psychology, College of Medical, Veterinary and Life Sciences, University of Glasgow, Glasgow, UK

## Abstract

Mutations in the collagen genes *COL4A1* and *COL4A2* cause Mendelian eye, kidney and cerebrovascular disease including intracerebral haemorrhage (ICH), and common collagen IV variants are a risk factor for sporadic ICH. *COL4A1* and *COL4A2* mutations cause endoplasmic reticulum (ER) stress and basement membrane (BM) defects, and recent data suggest an association of ER stress with ICH due to a *COL4A2* mutation. However, the potential of ER stress as a therapeutic target for the multi-systemic COL4A1 pathologies remains unclear. We performed a preventative oral treatment of *Col4a1* mutant mice with the chemical chaperone phenyl butyric acid (PBA), which reduced adult ICH. Importantly, treatment of adult mice with the established disease also reduced ICH. However, PBA treatment did not alter eye and kidney defects, establishing tissue-specific outcomes of targeting Col4a1-derived ER stress, and therefore this treatment may not be applicable for patients with eye and renal disease. While PBA treatment reduced ER stress and increased collagen IV incorporation into BMs, the persistence of defects in BM structure and reduced ability of the BM to withstand mechanical stress indicate that PBA may be counter-indicative for pathologies caused by matrix defects. These data establish that treatment for COL4A1 disease requires a multipronged treatment approach that restores both ER homeostasis and matrix defects. Alleviating ER stress is a valid therapeutic target for preventing and treating established adult ICH, but collagen IV patients will require stratification based on their clinical presentation and mechanism of their mutations.

## Introduction

Diseases caused by mutations in extracellular matrix components are traditionally considered matrix diseases, but many mutations elicit intracellular consequences (1). This has generated increasing interest in delineating the role of these intracellular responses in pathology and their potential as a therapeutic target. The basement membrane (BM) is an extracellular matrix structure that provides structural support, compartmentalizes tissues and influences cell behaviour and signalling (2). Collagen IV is a major BM component, and in vertebrates, the genes COL4A1–COL4A6 encode six collagen IV alpha chains [α1(IV)–α6(IV)]. These alpha chains assemble into three networks, α1α1α2(IV), α3α4α5(IV) and α5α5α6(IV), of which is α1α1α2(IV) is the most widely expressed (3). *COL4A1/COL4A2* mutations cause a multi-systemic disorder encompassing cerebrovascular disease, including intracerebral haemorrhage (ICH), as well as eye and renal defects (4–7) including HANAC syndrome (8,9). The variability in clinical presentation and severity means not every patient develops eye or kidney pathology in addition to cerebrovascular disease (7,10). The identification of rare mutations in sporadic haemorrhaging (11,12), and that common *COL4A2* variants are a risk factor for deep ICH (13) and white matter hyperintensities (14) in the general population, underscore an important role for *COL4A1/COL4A2* in common cerebrovascular disease and ICH. ICH accounts for 15% of adult stroke and 50% of paediatric stroke (15), and besides generic risk-reducing approaches, there are no specific treatments.

Mouse models with *Col4a1* missense mutations such as *Col4a1^+/SVC^* are excellent tools to investigate therapeutic strategies. They display ICH (4,16,17), eye disease including anterior segment dysgenesis (ASD) (18–20) and renal disease including Bowman’s capsule defects and tubular dysfunction (18,21,22). *Col4a1^+/SVC^* mice harbour a glycine to aspartic acid mutation affecting a highly conserved glycine residue of the Gly-X-Y repeat in the collagen domain of α1(IV) (18,22), equivalent to the vast majority (> 85%) of *COL4A1/COL4A2* mutations.

There are no treatments for diseases caused by *COL4A1/COL4A2* mutations, and the disease mechanisms remain poorly understood. Endoplasmic reticulum (ER) stress has emerged as a potential disease mechanism in addition to matrix defects for mutations in matrix components including collagen (1). Besides ECM defects, collagen IV mutations can lead to intracellular accumulation of collagen IV and ER stress (17,18,23,24). Genetic evidence from a family with a *COL4A2* mutation indicated that ER retention of α1α1α2(IV) and subsequent ER stress is associated with porencephaly and ICH (25), which was supported by animal data (26). ER stress due to ER retention of secreted protein elicits the unfolded protein response that entails three signalling arms: cleavage of XBP1 by IRE-1, proteolytic cleavage of ATF6 and phosphorylation of eIf2α causing the upregulation of ATF4 (27). ER stress has been observed in a wide spectrum of diseases such as (cerebro) vascular, renal (28–30) and ocular (31), pathologies, including age-related disease (32), but for many of these disorders, including adult ICH, its potential as therapeutic target remain unclear. Interestingly, ER stress can be targeted using compounds including 4-sodium phenyl butyrate (PBA), an FDA-approved chemical chaperone (33).

Here, we set out to determine the efficacy of PBA as a treatment for adult *COL4A1/4A2* pathologies. Our data establish that reducing ER stress is a therapeutic avenue for preventing and treating established adult ICH, but is not for effective eye and renal pathologies and can be counter-indicative for pathologies due to BM defects as it reduces their ability to withstand mechanical stress. This highlights the need for patient stratification for such treatment approaches based on both their clinical presentation and knowledge of the underlying mechanism of their specific mutations.

## Results

### Targeting ER stress as a preventative treatment for ICH

**Figure 1:**
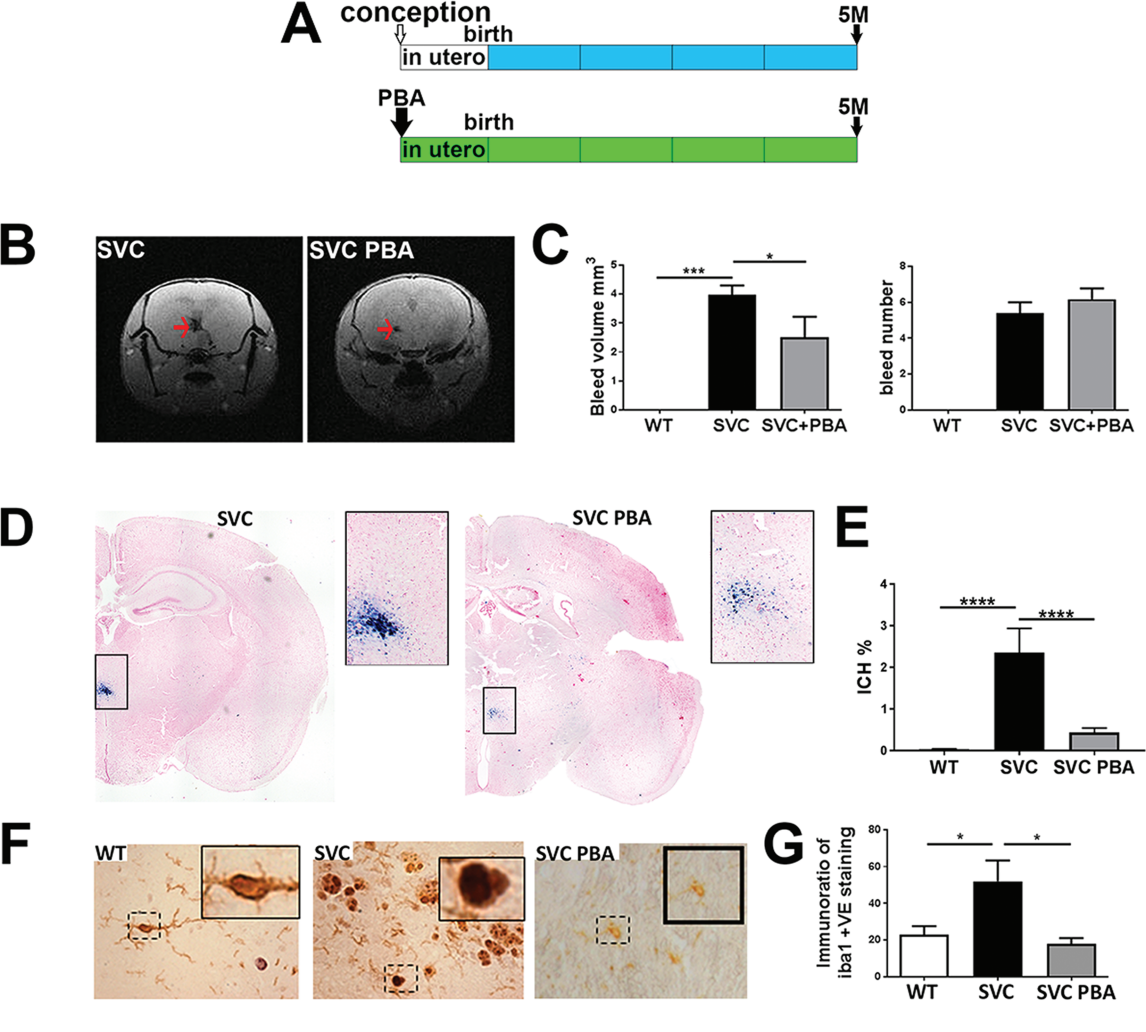
PBA reduces ICH. (**A**) Overview of preventative PBA treatment from conception to point of analysis. (**B**) MRI image of untreated and treated small with vacuolar cataracts (SVC) mice showing ICH (red arrow). (**C**) Image analysis of MRI data reveals reduced ICH bleed volume (left graph) but not ICH number of bleeds (right graph) (WT *n* = 6, SVC *n* = 10, SVC PBA *n* = 6). (**D**) Perl’s staining of brains from 5-month-old untreated *Col4a1^+/SVC^* and *Col4a1^+/SVC^* treated from conception (blue staining). (**E**) Image analysis of Perl’s staining ICH (WT *n* = 7, SVC *n* = 6, SVC PBA *n* = 12). (**F**) Immunostaining against Iba1 (brown) on brain sections with detail of dashed square provided in a small square. (**G**) ImageJ analysis of staining is provided in graph (WT *n* = 6, SVC *n* = 5, SVC PBA *n* = 3). One-way analysis of variance (ANOVA) with *post hoc* test [Bonferroni (G), Tukey (E); ^*^P-value < 0.05; ^***^P-value < 0.001].

As defects due to *COL4A1* mutations can present in childhood, a therapeutic approach will likely be long-term and would preferentially be orally administered. To model this preventative treatment, we treated *Col4a1^+/SVC^* mice (18,34) orally with PBA from conception, by treatment of pregnant dams, and ICH was determined in 5-month-old adult mice, treated daily (Fig. 1A). Magnetic resonance imaging (MRI) of the midbrain revealed that most ICH is centred towards the basal ganglia and, importantly, a ∼50% reduction in bleed volume of PBA-treated *Col4a1^+/SVC^* mice (Fig. 1B and C). As PBA did not affect the number of haemorrhages, this reduced severity is obtained by modulating bleed volume (Fig. 1C). Histopathological analysis using Prussian blue stain for hemosiderin (Perl’s staining) confirmed the MRI data (Fig. 1D and E). *Col4a1^+/SVC^* display increased levels of Iba1, a marker for neuroglial activation that is the initial step in the central nervous system inflammatory response following stroke (35), which was reduced in treated mice (Fig. 1F and G), supporting reduced cerebrovascular disease severity and neuroinflammation. Tail cuff plethysmography revealed no significant change in systolic blood pressure in mutant mice (Supplementary Material, Fig. S1), indicating the reduction in ICH is not dependent on altered vascular haemostasis.

### Modification of eye and renal defects

**Figure 2:**
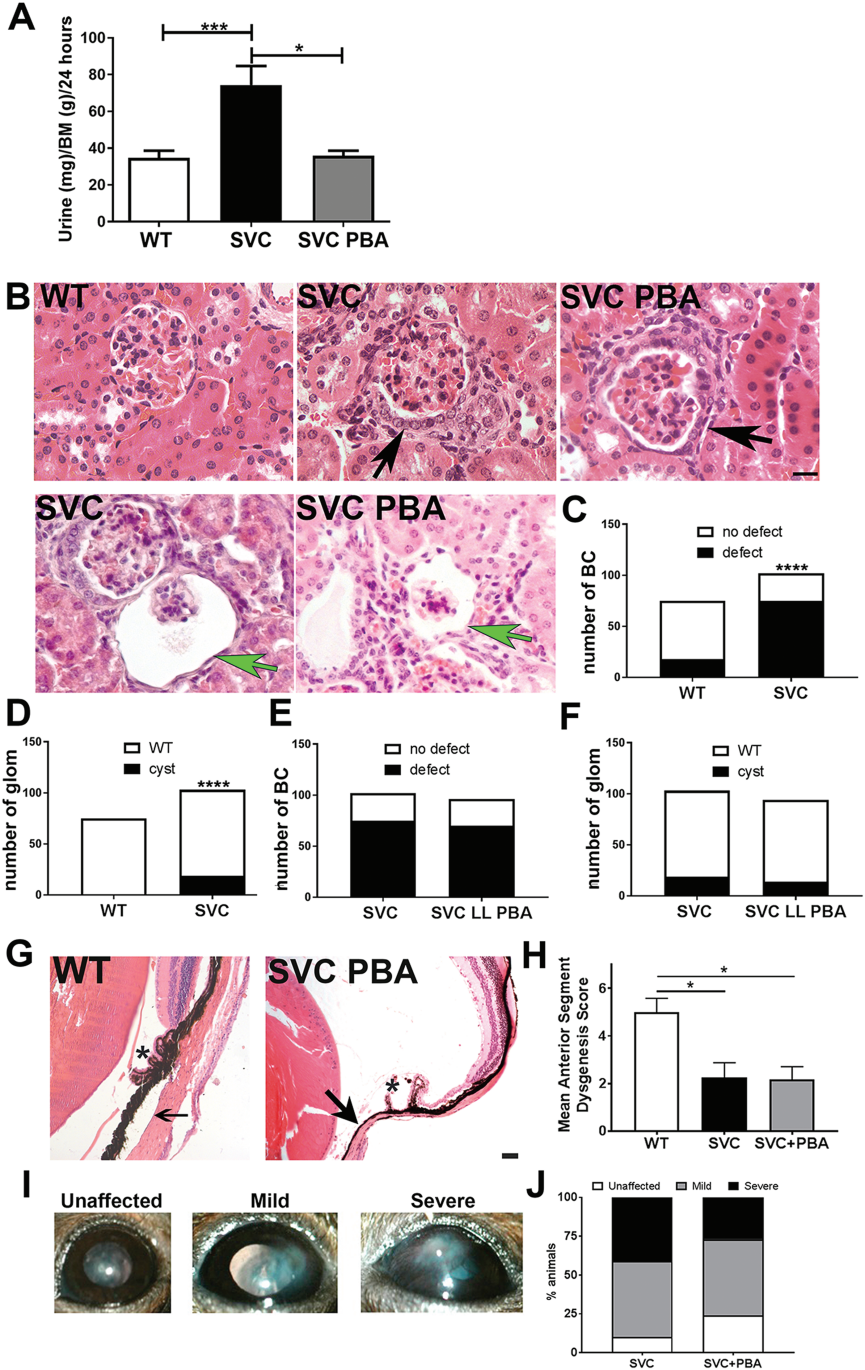
Effect of PBA treatment on adult eye and renal defects. (**A**) Daily urine output of five-month-old untreated WT littermate, untreated *Col4a1^+/SVC^* mice and treated *Col4a1^+/SVC^* (WT *n* = 20, SVC *n* = 18, SVC PBA *n* = 6). (**B**) Haematoxylin and eosin staining of kidneys revealed in untreated *Col4a1^+/SVC^* (SVC) and PBA-treated *Col4a1^+/SVC^* mice (SVC PBA) defects (cuboidal appearance, formation of multiple cell layers) of parietal epithelial cells of Bowman’s capsule (black arrow) and glomerulocystic kidney disease (retraction of vascular tuft, green arrow). SVC: untreated *Col4a1^+/SVC^*. (*n* = 4). (**C**) Image analysis of Bowman’s capsules in WT (*n* = 75 capsules) and *Col4a1^+/SVC^* (*n* = 102) (χ^2^ = 42.52; 1 df). (**D**) Image analysis of glomerulocystic kidney disease in WT (*n* = 75 glomeruli) and *Col4a1^+/SVC^* (*n* = 102) (χ^2^ = 15.49; 1 df). (**E**) Image analysis reveals similar frequency of Bowman’s capsule defects in treated (*n* = 84) and untreated (*n* = 102) *Col4a1^+/SVC^*. (χ^2^ = 0.009; 1 df) (**F**) Image analysis reveals similar frequency of cystic defects in treated (*n* = 103 glomeruli) and untreated (*n* = 94) *Col4a1^+/SVC^* (χ^2^ = 0.44; 1 df). (**G**) Haematoxylin and eosin staining of eyes revealed persistence of ASD defects in treated mice (SVC PBA) including iridocorneal adhesion (arrow), atrophy of the ciliary body (^*^) size bar = 50 μm. (**H**) Scoring of anterior segment (presence/absence of iridocorneal adhesion, ciliary body morphology, presence/absence of open Shlemm’s canal) in WT, untreated (SVC) and treated mutant (SVC + PBA) mice. (WT *n* = 4, SVC *n* = 4, SVC PBA *n* = 6 mice). (**I**) Slit lamp analysis revealed persistence of opacity of the cornea (scoring system applies only to corneal opacity) and iris hypoplasia in treated mice. Corneal neovascularization (see `severe’ panel) is also observed. The right panel is the graphical representation of the scoring of corneal opacity. (SVC *n* = 6, SVC PBA *n* = 4 animals). (**J**) Image analysis of corneal opacity scoring. (C)–(F): Two-sided chi-square test; (A), (H): One-way ANOVA *post hoc* Sidak test ^*^P-value<0.05, ^****^P-value <0.0001.

Col4a1 renal disease encompasses glomerular defects including hypertrophy of Bowman’s capsule and glomerulocystic kidney disease (18,21,22), as well as tubular defects that are associated with polyuria (increased urine production) (22). Preventative PBA treatment reduced polyuria (Fig. 2A). However, both untreated and treated mice displayed defects of the parietal epithelium in Bowman’s capsule with a cuboidal appearance of epithelial cells, suggestive of epithelial cell activation (21) (Fig. 2B), and/or formation of multiple cell layers (18,21,22): ∼74% (75/102) and ∼73% (70/96) of Bowman’s capsules of untreated and treated mice, respectively (Fig. 2B, C and E). Col4a1 glomerulocystic kidney disease includes retraction of the capillary tuft (21), which was detected in ∼18% (19/103) of glomeruli in untreated mice and ∼15% (14/94) of treated mice (Fig. 2B, D and F). The occurrence of capillary tuft retraction in *Col4a1^+/SVC^* appears to occur in glomeruli that do not appear to exhibit the parietal epithelial cell defect (Fig. 2B). Evidence of atrophy of the medulla also remained (Supplementary Material, Fig. S2B). These data indicate a differential response whereby Bowman’s capsule and glomerular defects are recalcitrant to PBA treatment while polyuria appears reduced.

In the eye, *Col4a1* mutations can lead to ASD encompassing corneal clouding, cataracts, iris hypoplasia and buphthalmos (enlargement of the anterior chamber) on slit lamp examination (Fig. 2I) (5,18,20). PBA-treated and untreated mice showed iris–corneal adhesions, dysgenic ciliary body and reduced or absence of trabecular meshwork (Fig. 2G and H). Severity scoring revealed PBA treatment did not alter ASD severity (Fig. 2H), while slit lamp analysis revealed iris hypoplasia, corneal clouding and neovascularisation in treated and untreated mice (Fig. 2I and J). The inner and outer retinal nuclear layers in *Col4a1^+/SVC^* can appear disorganized (Supplementary Material, Fig. S3B) and optic nerve cupping remains present following PBA treatment (Supplementary Material, Fig. S3D).

### Investigating the efficacy of PBA treatment on established phenotypes

**Figure 3:**
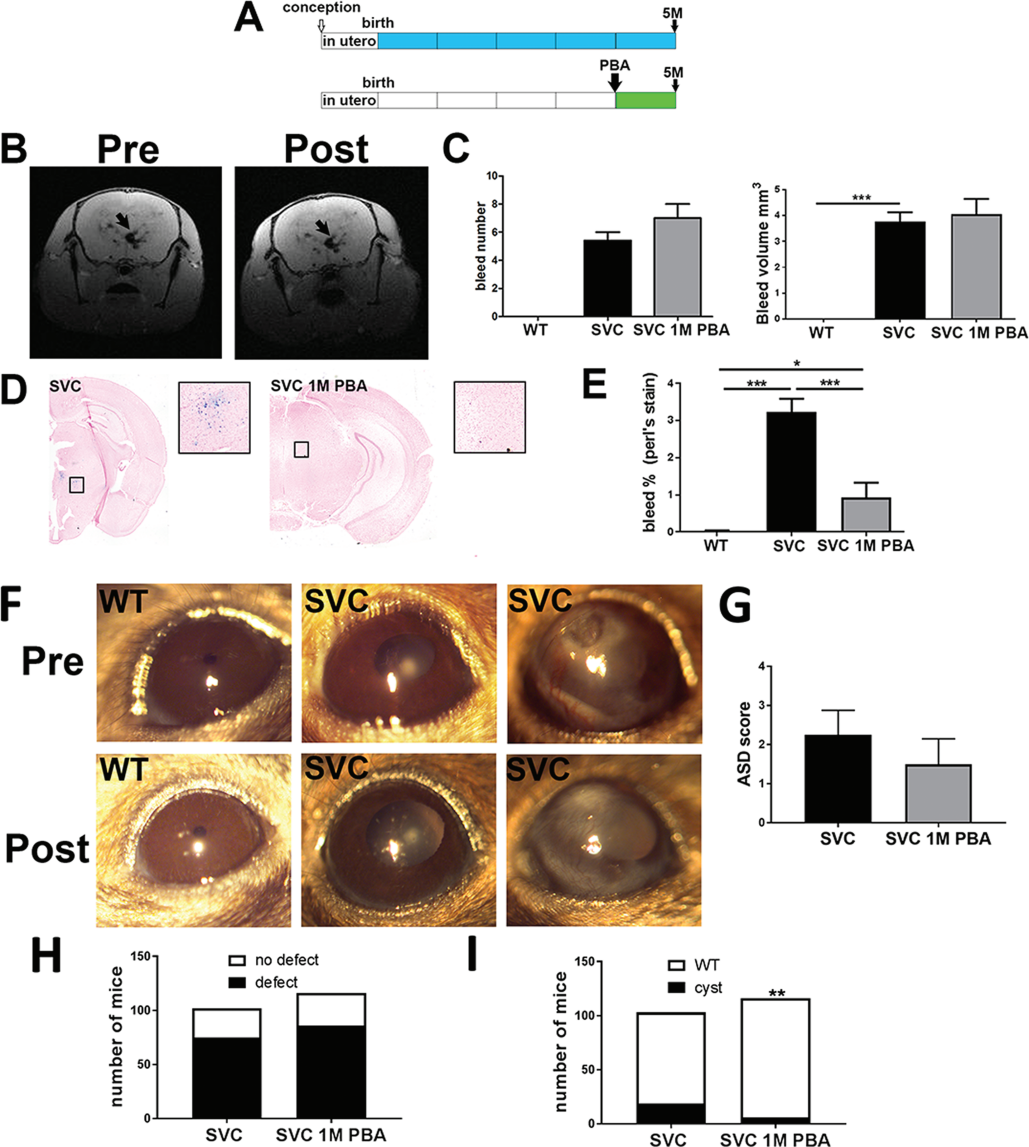
PBA treatment of established disease. (**A**) Diagram of 1-month oral PBA treatment for the treatment of established phenotypes in 5-month-old mice. (**B**) MRI image of a mutant animal pre and post treatment reveals ICH (black arrow). (**C**) ImageJ analysis of MRI data based on bleed number (top) and bleed volume (bottom) (SVC *n* = 11, SVC PBA *n* = 8). (**D**) Perl’s staining of brains sections reveals ICH (blue staining) untreated and treated mutant mice (SVC and SVC 1 M PBA). (**E**) Image analysis indicating percentage of tissue that stained positive for ICH (*n* = 3). (**F**) Slit lamp analysis reveals persistence of corneal opacity and iris hypoplasia. The images are of the same eye of the animal pre and post treatment (*n* = 4 animals). (**G**) Scoring of anterior segment (presence/absence of iridocorneal adhesion, ciliary body morphology, presence/absence of open Shlemm’s canal) in untreated (SVC) and treated *Col4a1^+/SVC^* (SVC 1M PBA). (SVC *n* = 4, SVC 1M PBA *n* = 4 mice) (**H**) Image analysis of Bowman’s capsule defects in untreated and treated mice (*n* = 116). (**I**) Image analysis of glomerulocystic defects (χ^2^ = 9.506; 1 df, *P* = 0.002). (Histopathology of the eye and kidney is provided in Supplementary Material, Figs S2 and S3). (D), (E) One-way ANOVA with Bonferonni *post hoc* test; (F) Two-sided chi-square ^*^P-value < 0.05, ^***^P-value < 0.001.

Treatment from conception or birth provides a proof-of-principle preventative approach that would only be possible for some familial cases. In most cases treatment will occur post ICH and molecular diagnosis. We therefore treated a 4-month-old *Col4a1^+/SVC^* for 1 month to determine if PBA can reduce established disease (Fig. 3A). While ICH severity was maintained on MRI analysis (Fig. 3B and C), ICH was significantly reduced based on histopathology using Perl’s stain (Fig. 3D and E). It should be taken into account that the resolution of our MRI analysis is not sufficient to detect microbleeds. Therefore, our data establishes that PBA can reduce established adult cerebrovascular phenotypes and suggests that PBA mainly affects small microbleeds not detectable with MRI.

Slit lamp analysis and histopathology did not reveal amelioration of ASD following treatment (Fig. 3F and G). Histopathological analysis of the retina did show a trend towards reduced optic nerve `cupping’, which in *Col4a1* mutant mice reflect optic nerve hypoplasia (23), but signs of retinopathy were also observed (Supplementary Material, Fig. S3C and D). In the kidney, defects in the parietal epithelium of Bowman’s capsule occurred in ∼74% (86/116) Bowman’s capsules (Fig. 3H), and cystic changes in ∼5% (6/116) glomeruli (Fig. 3I), compared to 74% and 18% in untreated mice, while polyuria (Supplementary Material, Fig. S2) was also observed. Thus, in our hands PBA has very limited efficacy for eye and glomerular renal phenotypes both as a preventative measure and treatment approach.

### PBA reduces ER stress and increases deposition of collagen IV in the BM

**Figure 4:**
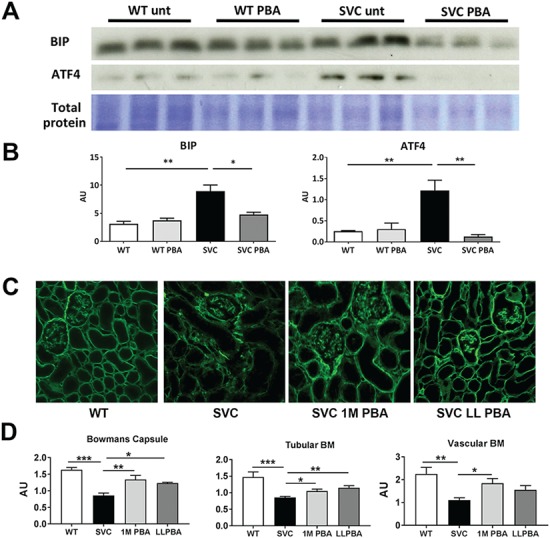
Chemical chaperone activity increases deposition of collagen IV *in vivo*. (**A**) Western blotting showed increased levels of Bip (∼2.9-fold increase) and ATF4 (∼4.6-fold increase) in untreated Col4a1^+/Svc^ (SVC unt) which is reduced following PBA treatment. Representative band of total protein stain is given as loading control (the entire gel is provided in Supplementary Material, Fig. S9). (**B**) ImageJ densitometry analysis of Bip and ATF4 (*n* = 3). (**C**) Immunostaining against Col4a1 in PBA-treated and untreated Col4a1^+/Svc^ mice (WT: wild-type; SVC: untreated; SVC 1M PBA: 1-month treatment; SVC LL PBA: lifelong chronic treatment from conception) on kidney sections revealed increased deposition of Col4a1 following PBA treatment. (*n* = 3–5 mice, Bowman’s capsule: SVC *n* = 30, SVC 1 M PBA *n* = 42, SVC LL PBA *n* = 16; Tubular BM SVC *n* = 89, SVC 1M PBA *n* = 77, SVC LL PBA *n* = 47; Vascular BM SVC *n* = 36, SVC 1M PBA *n* = 23, SVC LL PBA *n* = 15). (**D**) ImageJ analysis of fluorescence staining. (B), (D) One-way ANOVA *post hoc* Sidak test ^*^P < 0.05, ^**^P < 0.01, ^***^P < 0.001.

To determine if PBA was able to reduce ER stress levels *in vivo*, we assessed protein levels of ER stress markers Bip and Atf4 in kidney of adult *Col4a1^+/SVC^* mice treated from conception, which revealed a significant reduction in their levels (Fig. 4A and B). Reduced ER stress was also observed in mice treated for 1 month (Supplementary Material, Fig. S4). To further illuminate the mechanism of PBA we performed immunostaining against Col4a1 on kidney sections as kidney contains vascular, epithelial and glomerular BMs (GBMs). Both lifelong and 1-month PBA treatment increased staining of Col4a1 within the BMs (Fig. 4C and D), indicating that the reduced ER stress is coupled with increased secretion and staining of collagen IV in the BM.The absence of increased staining of collagen IV in lifelong versus 1-month treatments also suggests that long-term chemical chaperone treatment may not have additional long-term improvement/restoration of collagen IV deposition within the BM compared to a shorter treatment. Besides being a chemical chaperone PBA can also inhibit histone de-acetylation activity, which could lead to the increased transcription of collagen IV. Quantitative reverse trancription polymerase chain reaction (qRT-PCR) analysis on mRNA samples from kidneys from untreated and lifelong treated mice revealed that PBA did not alter collagen IV mRNA levels (Supplementary Material, Fig. S5), supporting that the increased collagen IV staining is due to the chemical chaperone activity of PBA.

### Effects of chemical chaperone on BM structure function

**Figure 5:**
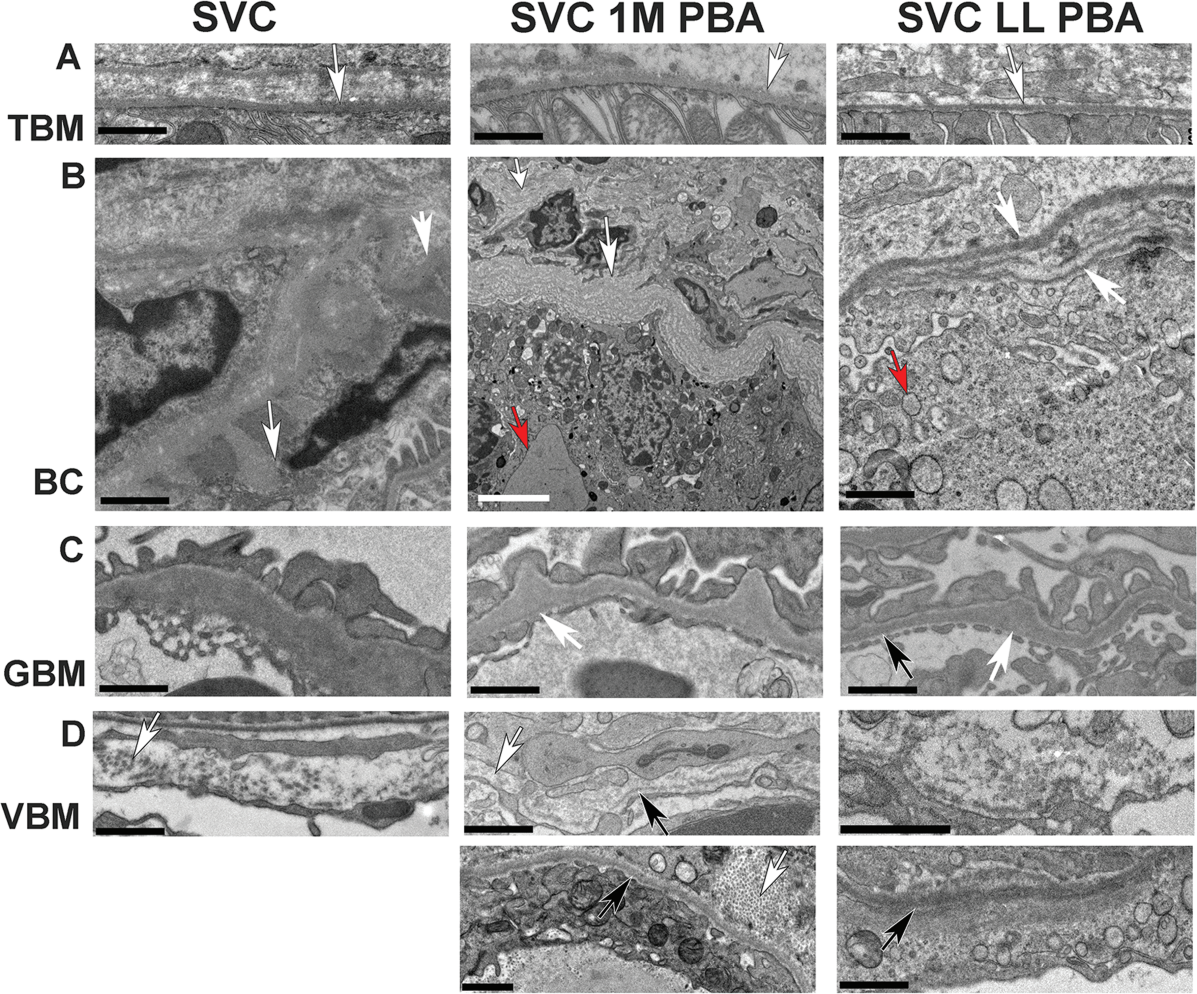
Effect of PBA on BM structure. (**A**) Normal appearance of BM of tubules (white arrow) in untreated *Col4a1^+/SVC^* (SVC) and mice treated for 1 month at 4 months of age (SVC 1M PBA) or from conception for 5 months (SVC LL PBA). (**B**) Severe defects in BM of Bowman’s capsule in all mice including bulges (white arrow SVC), basket weave appearance (white arrow SVC 1M PBA) and multiple layers (white arrow SVC LL PBA). Evidence of enlarged ER (red arrow SVC 1M PBA) and increased vesicles (red arrow SVC LL PBA) is also observed. (**C**) Irregular thickening (white arrow) of GBM in treated and untreated mice. Thinner BM areas are also observed (black arrow). (**D**) VBM defects include interruption (white arrow SVC, 1M PBA), presence of collagen fibrils (white arrow bottom panel 1 M PBA) and more fuzzy but continuous BM (bottom panel LL PBA) black size bar 1 μm, white size bar 5 μm. One-way ANOVA *post hoc* Tukey test ^*^P < 0.05, ^**^P < 0.01, ^***^P < 0.001.

To determine if increased collagen IV deposition alters BM structure we performed transmission electron microscopy (EM) analysis of kidney, which enables analysis of vascular, endothelial and epithelial BMs. In wild-type (WT) mice, PBA did not alter collagen IV deposition (Supplementary Material, Fig. S6). In mutant mice, despite increased collagen IV staining (Fig. 4E), PBA did not affect mean thickness of renal and vascular BMs (VBMs) (Supplementary Material, Fig. S7A–D), but BMs were characterized by a large variation in thickness (Supplementary Material, Fig. S7C and D). EM analysis revealed that PBA treatment did not affect tubular BM structure (Fig. 5A), which is unaffected in *Col4a1^+/SVC^* mice (Fig. 5A) and (22). However, we did occasionally observe small focal duplications in some animals (1-month treatment: 1/4 animals; lifelong treatment: 2/5 animals) (Supplementary Material, Fig. S7E). Defects in BM of Bowman’s capsule included an irregular appearance, lamellation and multiple layers, as observed for untreated *Col4a1* mutant mice [Fig. 5B and (18,22)]. Although the overall reduction of ER stress levels was observed, some treated animals displayed more prominent ER vesicles in parietal epithelial cells of Bowman’s capsule (Fig. 5B). The GBM of treated and untreated animals was similar with irregular GBM thickness [Fig. 5C and (22)], but average thickness remained unchanged (Supplementary Material, Fig. S7C). However, PBA treatment appears to result in a reduction in the variability of GBM thickness (Supplementary Material, Fig. S7C). The vascular BM revealed heterogeneity whereby the BM in treated animals appeared in some sections more continuous compared to untreated mice (Fig. 5D). However, significant areas of apparent BM absence, disruptions and diffuse BMs with reduced electron density were also observed. Both treated and untreated *Col4a1^+/SVC^* mice showed fibrillar collagen deposition (Fig. **5**D). Taken together these data indicate that PBA treatment does not significantly improve BM ultrastructure.

**Figure 6:**
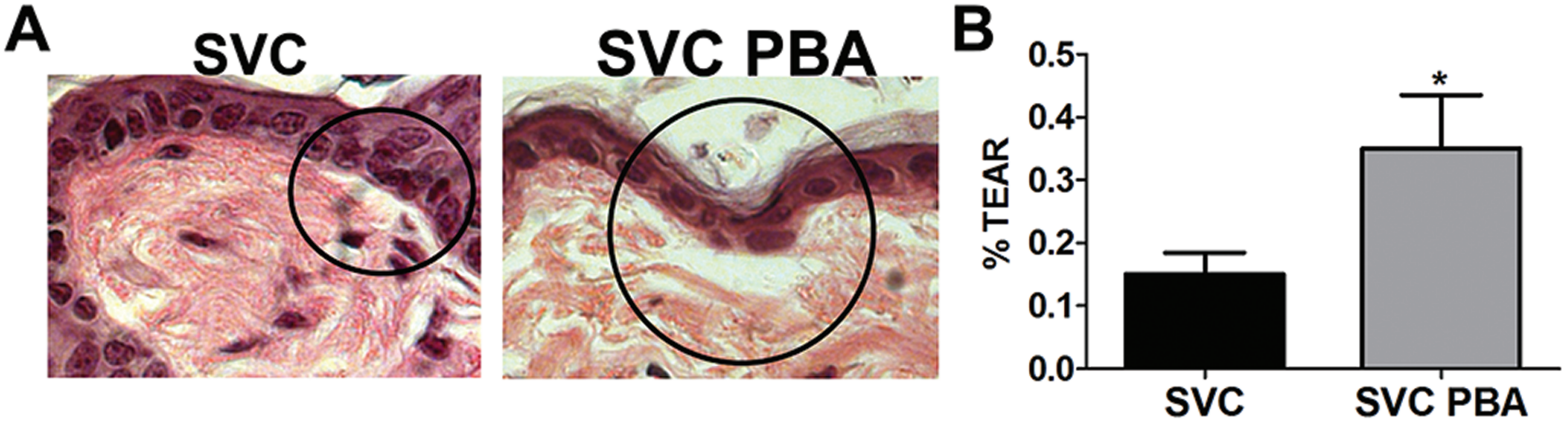
PBA does not improve BM strength. (**A**) PAS stain of tissue section from *Col4a1^+/SVC^* and 1-month treated *Col4a1^+/SVC^* (SVC PBA), which shows separation of dermis from epidermis (circle). (**B**) ImageJ analysis revealed increased separation in treated mice (SVC *n* = 7, SVC PBA *n* = 4) unpaired *t*-test ^*^P < 0.05 (Stain of WT littermate is provided in Supplementary Material, Fig. S8).

Given the increased deposition of collagen IV into the BM, we investigated if this affected BM strength. The dermal BM is critical for the adhesion of the epidermis to the dermis, and dermal BM defects cause blister formation in epidermolysis bullosa (36). Tape stripping on the back skin of mice generates mild mechanical stress, which enables a measurement of the ability of the BM to withstand separation of the dermis from the epidermis and blister formation (37). We employed this tape stripping procedure followed by periodic acid–Schiff (PAS) staining (Fig 6A) to assess the ability of the BM to withstand mechanical stress as a measure of its strength. Compared to WT, *Col4a1^+/SVC^* showed increased separation of dermis from the epidermis, indicating a weaker BM (Supplementary Material, Fig. S8). Surprisingly, PBA did not reduce the level of separation, but apparent increased severity was observed (Fig. 6B). These results provide evidence that increased collagen IV secretion in mutant mice and chemical chaperones can reduce the ability of BMs to withstand mechanical strength.

## Discussion

Disorders due to mutations in BM and matrix components are rare diseases and for most of these diseases treatment remains a long-term goal. Specific treatments are often also lacking for common diseases including haemorrhagic stroke that accounts for 15% of adult stroke and is a major burden on society (15). Developing specific or more effective treatments is underpinned by understanding disease mechanisms. Mutations in *COL4A1/COL4A2* cause ER stress and BM defects (10,22,25) and while ER stress occurs in a large number of diseases, including extracellular matrix disorders (1,38), the efficacy of targeting ER stress as a long-term therapeutic approach for adult onset diseases, either as preventative approach or as treatment of existing conditions, is an important question to address. Mutations in ECM and BM components most often result in complex multi-systemic diseases (36). Therefore, it is important to adopt a holistic approach and determine the efficacy of compounds across a spectrum of affected tissues.

We performed a chemical chaperone treatment to target ER stress in mice carrying a *Col4a1* glycine missense mutation, the most common mutation type identified in patients (39). While ICH due to *COL4A1/COL4A2* mutations can occur throughout life (4,6,17,19,36,40), many of the vascular and extra-vascular pathologies, including renal and eye defects, develop with age and present in adulthood in mice (18,22,41). We therefore performed a PBA treatment from conception to investigate if targeting ER stress can prevent adult disease. We focused on ICH as it underlies the cerebrovascular pathologies caused by *COL4A1/COL4A2* mutations, including porencephaly and small vessel disease (10). This revealed that an oral preventative PBA treatment reduces adult ICH due to *Col4a1* glycine mutations. These data are supported by a short term PBA treatment of newborn pups (26) and mice harbouring a very severe splice mutation (42). However, preventative treatment from birth or early childhood will likely only be possible for rare familial cases following molecular diagnosis/prenatal screening. For the majority of cases treatment will and can only commence after ICH and molecular diagnosis has occurred, which also applies to sporadic ICH in the general population for which collagen IV is a risk factor (11–14). Interestingly and importantly, treating adult mice for 1 month with PBA reduced total ICH, providing strong evidence that targeting ER stress using chemical chaperones has efficacy as a treatment for pre-existing ICH and COL4*A1*-associated cerebrovascular disease.

A core requirement of personalized medicine is to delineate patient groups for which a particular treatment is recommended and those for which alternative approaches are required. Investigating the mode of action of treatments in light of the pathomolecular disease mechanisms is therefore important. Besides a chemical chaperone PBA can also act as a histone deacetylase inhibitor (43). The absence of altered collagen IV mRNA levels provides evidence that PBA is acting through its chemical chaperone activity. In addition, although PBA has been shown to be able to reduce blood pressure in hypertension (44), it did not affect blood pressure in our model, indicating its effects on ICH is independent of blood pressure. PBA treatment reduced ER stress and increased deposition of collagen IV in BMs, which was associated with reduced mechanical strength of the BM. This increased secretion in response to PBA treatment *in vivo* is supported by data from other matrix diseases including Pierson syndrome due to laminin B2 mutations (29) and Osteogenesis Imperfecta caused by a *Col1a1* mutation (45). As our model is heterozygous for the causal mutation (18), similar to human patients (39), we were not able to determine if the secreted collagen is mutant or WT collagen. Similarly, the extent to which this additional secreted collagen IV is integrated into the BM remains to be determined. However, our data do indicate that promoting collagen secretion via chemical chaperone treatment is counter-indicative for COL4A1 pathologies caused by BM defects as PBA treatment reduced the ability of BM to withstand mechanical stress. In addition, the persistence of eye and renal defects indicate that chemical chaperone treatment represents a potential therapeutic approach for patients that present only with COL4A1-associated ICH but is not effective for those that also exhibit renal or eye disease, establishing a criterion for patient stratification. The apparent lack of effectivity for the eye phenotype may be related to the avascular nature of the adult lens, which plays a key role in Col4a1 eye disease (46), as previous data in cartilage (47) suggested that PBA is not effective for avascular tissues. It will now also be important to establish if the lack of effectivity for Col4a1 renal disease also applies to other kidney pathologies due to mutations in BM components such as Alport syndrome, where PBA reduced ER stress in cells with *COL4A5* missense mutations (48).

The tissue-specific outcomes of our treatment also illustrate the urgent need to increase our understanding of the molecular disease mechanisms. Our previous work in the kidney showed that *Col4a1* Bowman’s capsule defects were associated with matrix defects and the tubular disease with ER stress as it occurred in the absence of BM defects and was associated with chronic ER stress levels (22). The involvement of ER stress in the eye disease has remained unclear with apparent conflicting data (46,49). Given the reduced ER stress and ICH following treatment, and persistence of matrix defects and kidney pathology, these data provide significant evidence to the hypothesis that collagen IV mutations may elicit cell and/or tissue-specific responses and disease mechanisms, with varying contributions of ER stress and/or matrix defects. In this case, it is tempting to suggest a major role of matrix defect in the Bowman’s capsule pathology and ER stress in cerebrovascular disease. However, while many *COL4A1/COL4A2* mutations in mice and patients cause matrix defects and intracellular retention (7,18,25), not all mutations elicit the same cellular response (24). For example, mice homozygous for the *Col4a1* G498V mutation, detected in HANAC patients, are viable (21) in contrast to other glycine mutations in mice (4,18,19), while other non-glycine mutations do not appear to cause ER stress (24) and presumably act though matrix defects. Given that our data lead us to suggest that chemical chaperone treatment may not be warranted for *COL4A1/4A2* mutations that act via matrix defects and do not cause ER stress, it is important to delineate the actual contribution of intra- and extracellular sequelae to disease pathogenesis and confirm treatment efficacy across different *Col4a1*/*Col4a2* mutations.

In conclusion, our data establish that reducing ER stress is a therapeutic avenue for preventing and treating established adult ICH but can be counter-indicative for pathologies due to extracellular matrix defects as it reduces their ability to withstand mechanical stress. This highlights the need for patient stratification for such treatment approaches based on both their clinical presentation and knowledge of the underlying mechanism of their specific mutations.

## Materials and Methods

Animal studies were performed in accordance with UK Home Office regulations (Project license 70/8604). PBA (1 g/kg/day) (33) was administered orally via drinking water (lifelong treatment), or via gavage (1-month treatment). Animals were randomly allocated to treatment/no-treatment group before genotyping and development of overt phenotypes. Samples were labelled numerically and did not display treatment/genotype status, blinding the researcher. Un-blinding occurred following completion of datasets.

### Metabolic cage studies

Animals were individually housed and allowed to acclimatize (24 h). Daily urine samples were collected and water consumption measured.

Tail plethysmography was performed as previously described (17).

Slit lamp analysis was performed as described (18).

### MRI Analysis

MRI scanning was performed on a 7Tesla Bruker Biospec scanner (Bruker, Coventry, UK). A 72 cm volume resonator birdcage coil was used for transmitting and four-channel phased array surface coil for receiving the MRI signal. A gradient echo imaging sequence was used to acquire T_1_ weighted images, with the following parameters: 1.76 cm × 1.76 cm Field of View, 176 × 176 matrix, 100 μm × 100 μm, 14 coronal slices, thickness 0.5 mm, repetition time 200 ms, echo time 3.3 ms, 8 averages, flip angle 30°, total scan time 4 min 41 s. The 14 slices were acquired back from the rhinal fissure, excluding the olfactory bulb and cerebellum. Images were acquired and reconstructed using Bruker Paravision 5.1 software. Images were analyzed using ImageJ software to calculate bleed volumes and number of bleeds.

Dermal BM analysis was performed by repeated application and removal of autoclave tape on shaven dorsal skin. Following dissection of skin areas, samples were processed for histology. ImageJ was used to measure ratio of basement separation versus length of epidermis, excluding hair follicles.

### Histopathology

Tissues were fixed (10% neutral buffered formalin or 4% paraformaldehyde) and paraffin embedded. Sections were stained with haematoxylin–eosin or Perl’s Prussian blue using standard protocols. For Perl’s Prussian blue staining to assess ICH, image analysis was performed using ImageJ, colour threshold plugin (50) to calculate the area of the section stained positive for hemosiderin. A ratio of the bleed area versus tissue section area represents bleed percentage. For each brain, six sections of the midbrain, separated by 250 μm, were analysed and the total area of the section stained positive for hemosiderin was determined. Skin sections were stained with PAS using standard protocols. Histopathology of the eye and kidney section was performed using haematoxylin–eosin staining. The eye section was scored based on (20). Briefly, scoring of anterior segment defects was based on the presence/absence of iridocorneal adhesion, ciliary body morphology (with three to five foliations being considered normal), and presence/absence of open Shlemm’s canal. Absence of iridocorneal adhesion, three to five foliations of the ciliary body and presence of open Shlemm’s canal were given a score of 1 whereas defects were scored as 0. Mean ASD score was calculated per animal with a maximum score of 6. Kidney sections were scored for the presence of defects in Bowman’s capsule (including cuboidal appearance of parietal epithelial cells, formation of multiple cell layers) and glomerulocystic kidney disease (retraction of the glomerular tuft, dilation of Bowman’s Space, circumference of Bowman’s capsule measured using the segmented line tool in ImageJ) as described (21). Statistical analysis was performed on glomerulus/Bowman’s capsule data from > 10 random images of three sections per animal of four mice.

***EM analysis.*** Tissues were fixed in 2% glutaraldehyde in phosphate buffer and processed as described (34). EM thickness was measured as described (22): measurements were taken every ∼800 nm in at least three animals with three to five structures (e.g. Bowman’s capsule) analysed per animal. Statistical analysis was performed using averages of individual structures.

qRT-PCR analysis was performed as previously described (17). mRNA extracts were prepared using Tri Reagent followed by cDNA synthesis using Affinity Script cDNA Synthesis Kit as per manufacturer instructions. RT-PCR was performed in triplicate using Power SYBR Green PCR Master Mix (Thermo Fisher Scientific, Loughborough, UK) as per manufacturer instructions. Analysis of mRNA levels was performed using the ΔCT method and was corrected for 18S ribosomal RNA.

### Immunoblotting

Protein extracts were prepared in RIPA buffer containing protease (Roche Applied Science, Welwyn Garden City, UK) and phosphatase inhibitors (PhosStop Roche). Membranes were blocked with 5% milk or bovine serum albumin (BSA) before incubation with primary antibodies [BIP (1/10,000, BD Transduction, Biosciences, Wokingham, UK), ATF4 (1/2500 Santa Cruz, Heidelberg, Germany)], horseradish peroxidase conjugated secondary antibodies (GE Healthcare, Little Chalfont, UK) and development using chemiluminescense (Millipore, Watford, UK). Protein levels were corrected for Coomassie staining of total protein gels ran or protein stain on membrane (Memcode, Pierce, Thermo Fisher). Densitometry was performed using ImageJ.

### Immunohistochemistry

Staining against collagen IV (H22 for Col4a2; 1/100) on cryosections was performed as described (22,34). Paraffin-embedded sections underwent antigen retrieval using citrate buffer, were incubated with primary antibodies against IBA1, then counterstained using Impress DAB kit (Vector Laboratories, Peterborough, UK). Images were captured using a Axiocam microscope and Zen (Zeiss, Cambridge, UK) imaging software.

Statistical analysis (Graphpad Prism) was done by unpaired student’s *t*-test, chi-square or one-way ANOVA with *post hoc* analysis.

## Supplementary Material

Supplementary Data
