# Supplementary material for: 4-Sodium phenyl butyric acid has both efficacy and counter-indicative effects in the treatment of Col4a1 disease
Source: Hum Mol Genet. 2018 Oct 22;28(4):628–38. doi: 10.1093/hmg/ddy369 (PMC6360271; doi:10.1093/hmg/ddy369)
Supplement: Supplementary Data [file ddy369_supp.docx]

**Supplemental Figures**

**Supplemental Figure 1**

**
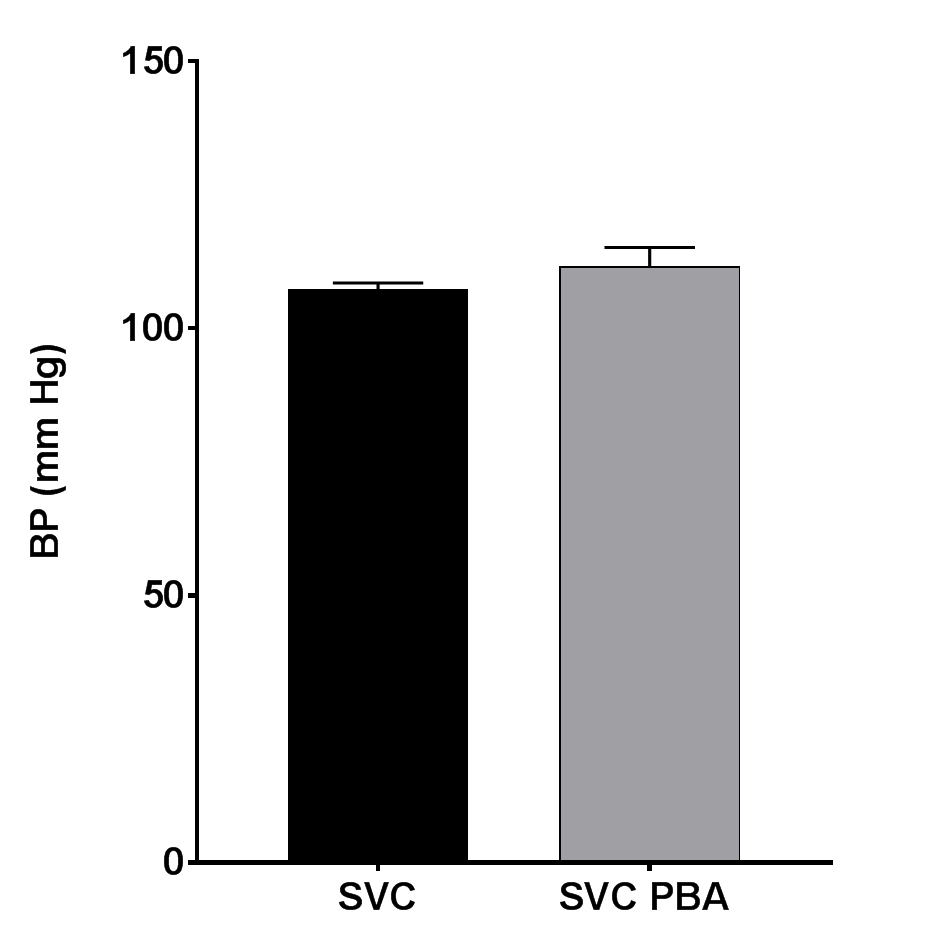
**

Analysis of systolic blood pressure in untreated *Col4a1^+/SVC^* (SVC) and *Col4a1^+/SVC^* mice treated from conception with PBA (SVC PBA) suggest PBA does not reduce blood pressure (SVC n = 3, SVC PBA n = 6).

**Supplemental Figure 2**

**
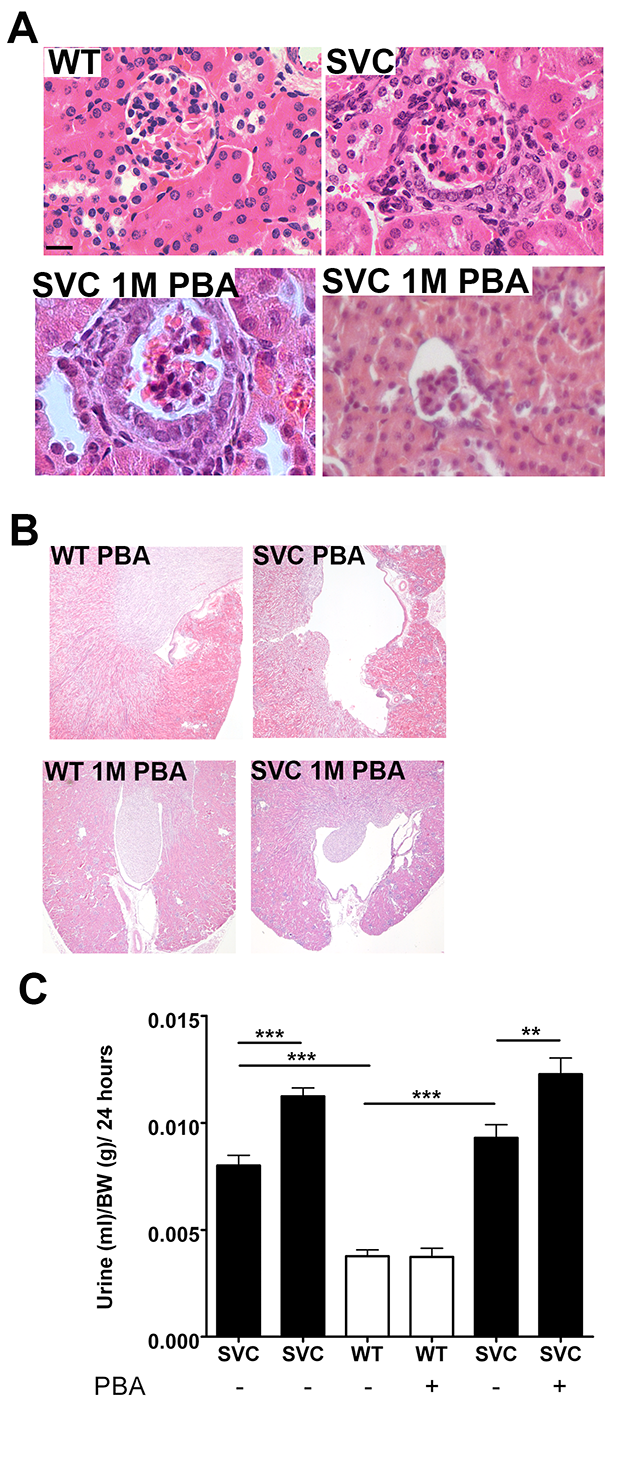
**

Analysis of kidney in mice treated for 1 month with PBA. (A) Histology of Bowman’s capsule defects and glomerulocystic kidney disease in 5 month old wild type (WT), untreated *Col4a1^+/SVC^* (SVC) and treated SVC mice (SVC 1M PBA) reveals persistence of hypertrophy of Bowman’s capsule with multiple layer of parietal epithelium. (B) Persistence of apparent atrophy of medulla in treated SVC mice treated either from conception (SVC PBA) or for 1 month (SVC 1M PBA) (black arrow). (C) Measurement of daily urine production (corrected per gram body weight) of mutant and wild type mice before and after 1 month PBA treatment. (n = 4)

**Supplemental Figure 3**

**
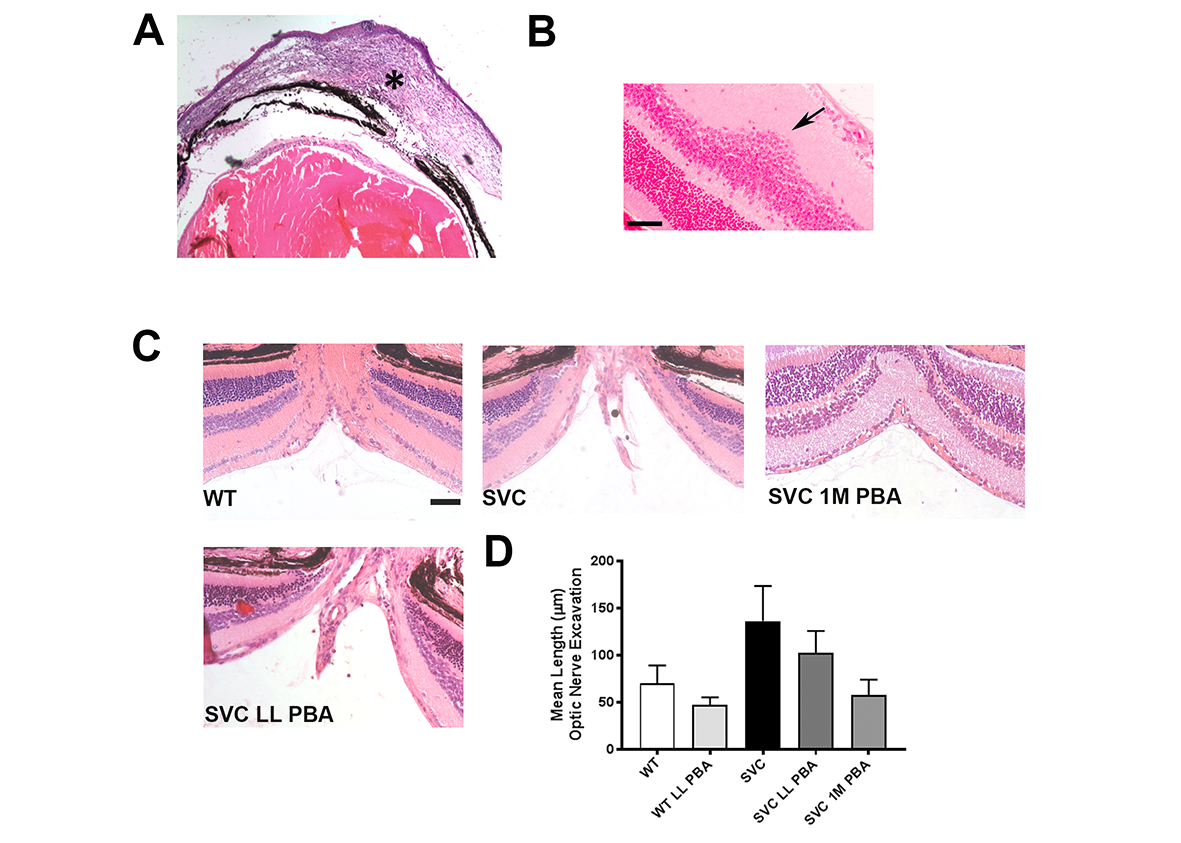
**

(A) Histopathology revealed corneal ulceration in PBA treated mice alongside irido-corneal adhesion. (B) Retinal defects observed in treated *Col4a1^+/SVC^* included irregular appearance of inner nuclear layer of retina. Size bar 50 µm. (C) Histopathologic analysis of optic nerve cupping in untreated Col4a1^+/SVC^ (SVC) and treated either for 1 month (SVC 1M PBA) or lifelong (SVC LL PBA). (D) Image J analysis of optic nerve cupping as assessed by measuring length of retraction of optic nerve from the retina. N=4-6.

**Supplemental Figure 4**

**
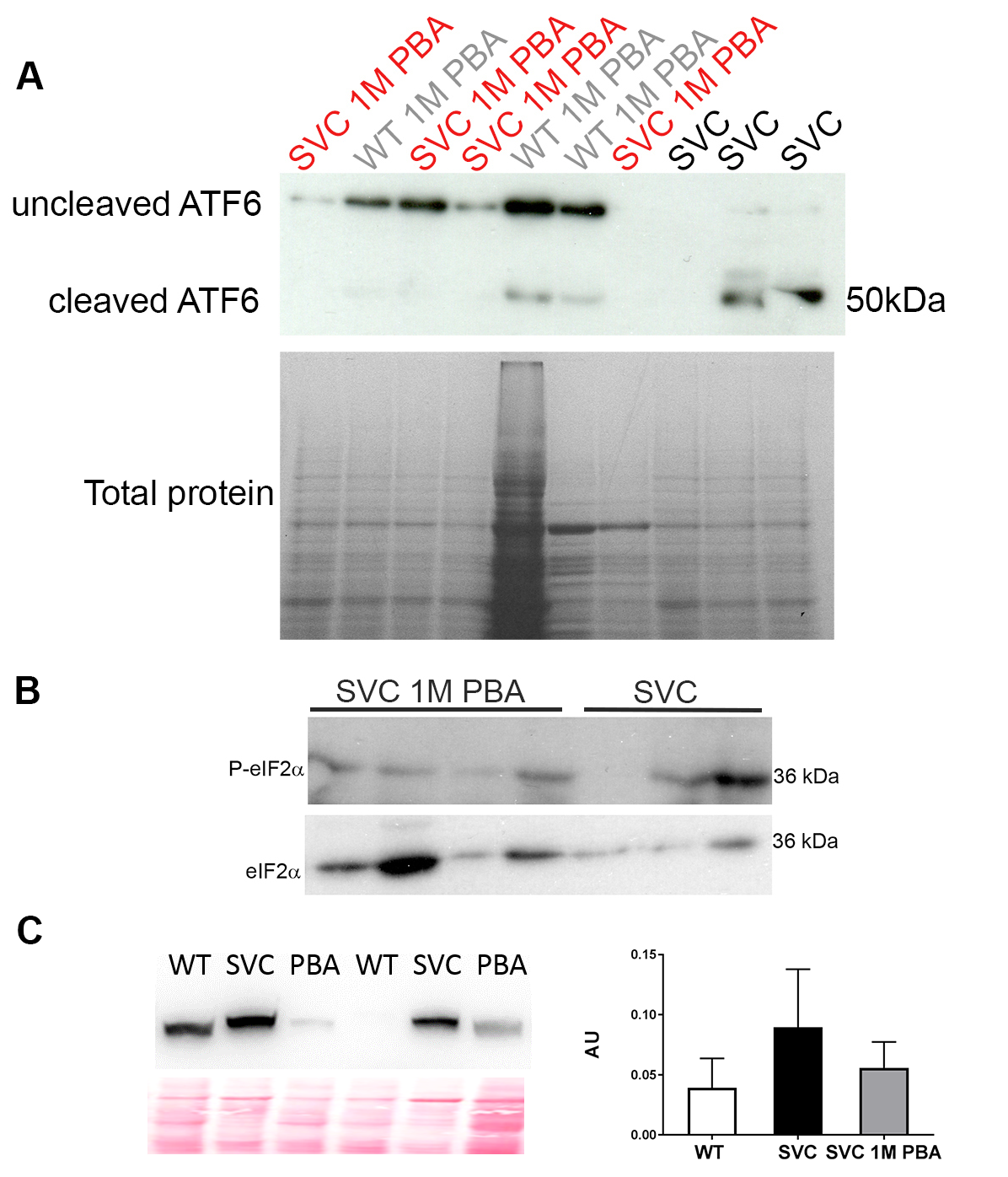
**

**ER stress in kidney of untreated and treated *Col4a1^+/SVC^* mice for 1 month with PBA. (A)** Western blotting against ER stress sensor ATF6. ER stress induces cleavage of the 90kDa inactive protein to release the active 50kDa fragment which acts as a transcription factor. PBA treatment (red lanes) results in an apparent reduction activated ATF6 compared to untreated SVC (black lanes). Total protein loading is provided as control. (B) PBA treatment reduced apparent ratio of phosphorylated eIf2α versus total eIf2α. (C) PBA treatment appears to reduce Bip protein levels (n=4).

**Supplemental Figure 5**

**
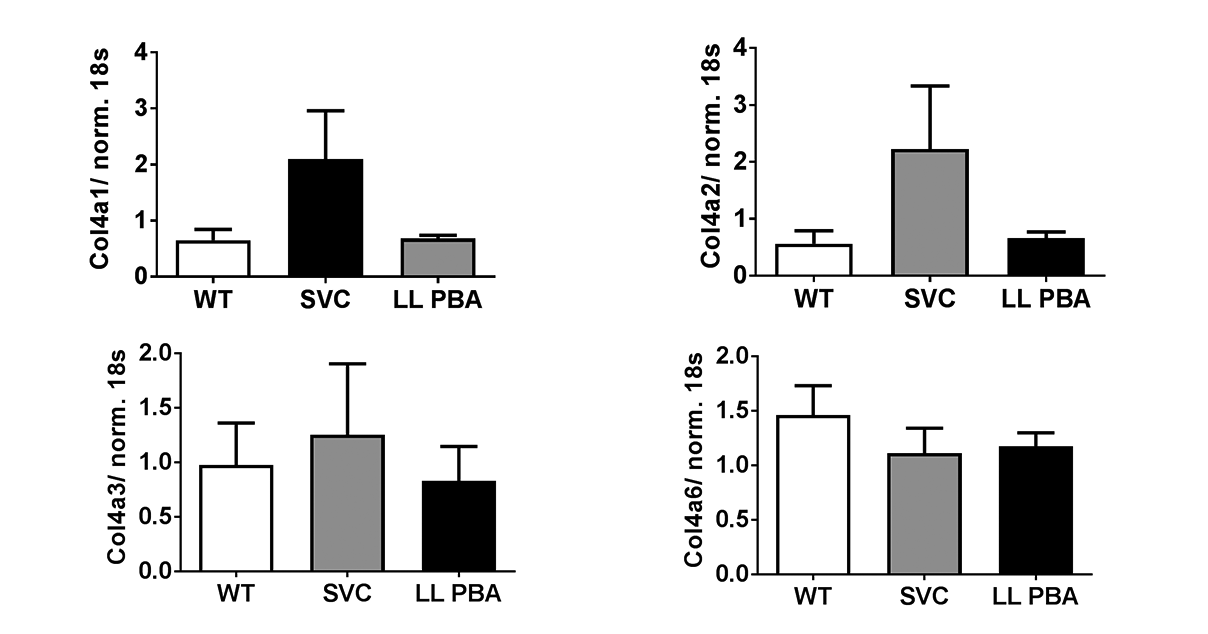
**

**PBA does not alter collagen IV mRNA levels.** Quantitative RT-PCR was performed on mRNA samples from kidneys of wild type (WT), untreated *Col4a1^+/SVC^* and *Col4a1^+/SVC^*  treated with PBA from conception (LL PBA). PBA does not increase collagen IV mRNA levels as indicated by Col4a1, Col4a2, Col4a3 and Col4a6 mRNA levels. (WT n = 3, SVC n=4, SVC LL PBA n = 4).

**Supplemental Figure 6**

**
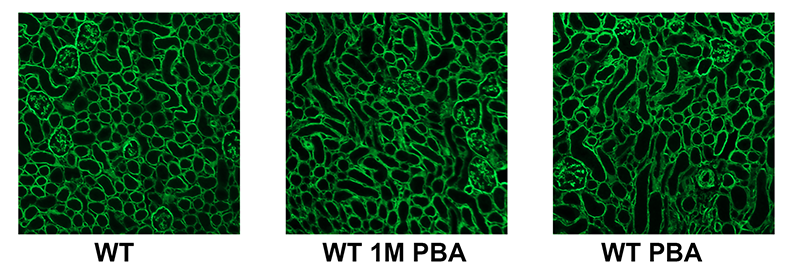
**

**Effect of PBA on Col4a1 deposition in wild type basement membranes.** Immunostaining against Col4a1 on kidney section of 5 month old untreated wild type mice (WT) and mice treated for 1 month from 4 months of age (WT 1M PBA) or from conception (WT PBA).

**Supplementary Figure 7**


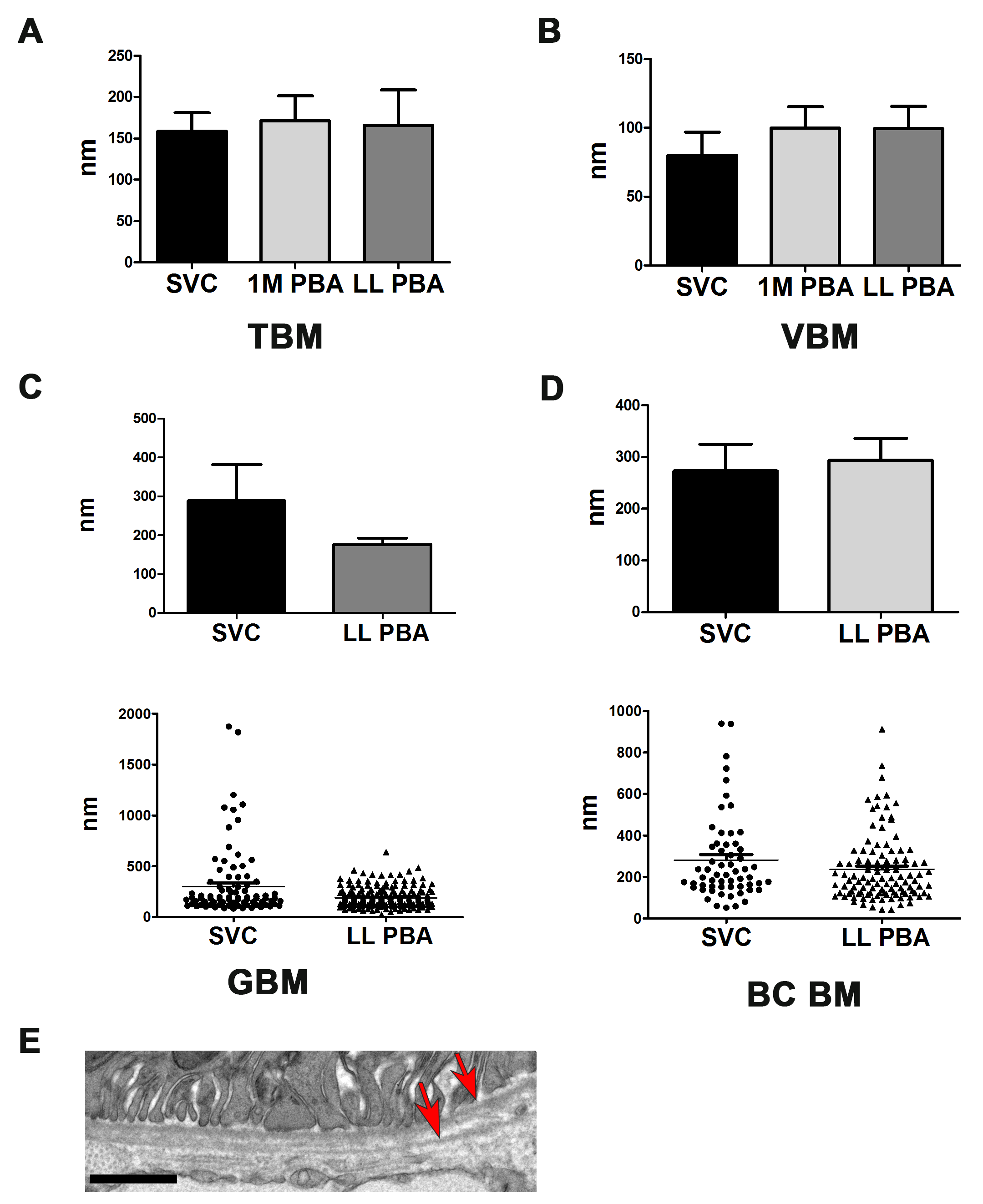


**Effect of PBA on basement membrane structure**. Image analysis of EM data of 5 month old Col4a1*^+/SVC^* (SVC) mice to assess average BM thickness (nm). Mice were either untreated, treated for 1 month from 4 months (1M PBA) or from conception (LL PBA). (A) tubular BM (B) Vascular BM (C) Glomerular BM with thickness of individual glomeruli given below indicating variability in thickness in untreated mice. (D) Average thickness of BM of Bowman’s Capsule with thickness of individual Bowman’s capsules given underneath. (E) Duplication of tubular basement membrane observed in PBA treated mice (red arrow). Size bar 1µm.

**Supplemental Figure 8**

**
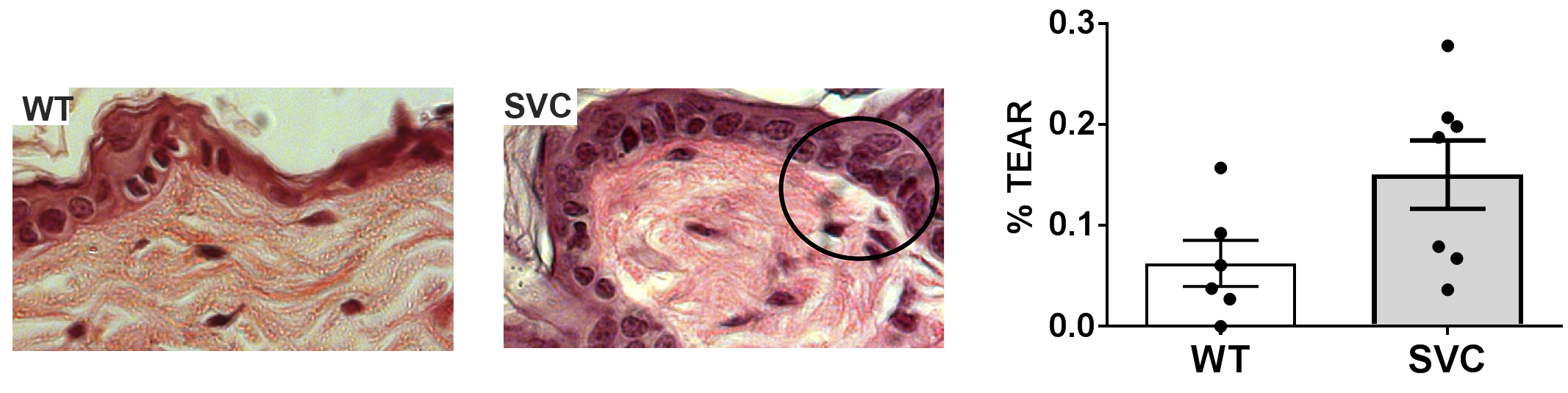
**

Blister formation in *Col4a1^+/SVC^* mice. Staining of skin section from wild type and *Col4a1^+/SVC^* mice that have undergone tape stripping showing increased detachment in mutant mice. (p = 0.06 t-test)

**Supplemental Figure 9**

**
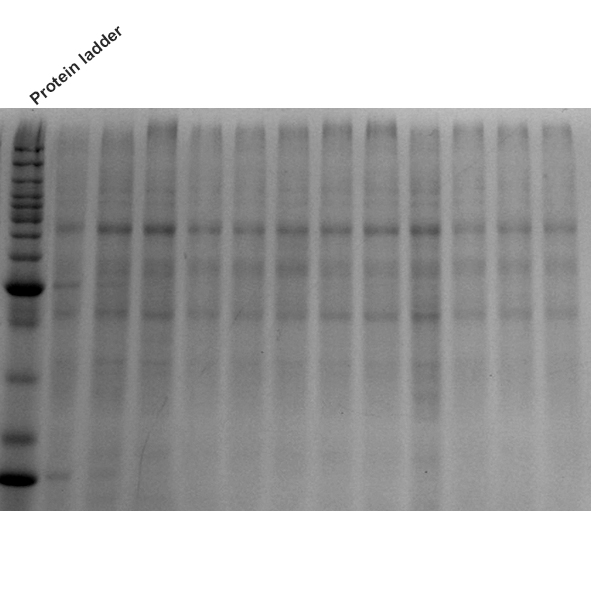
**

Protein loading gel from Figure 4.
